# Applications and benefits of the British Society for Antimicrobial Chemotherapy Resistance Surveillance Project—legacy and future

**DOI:** 10.1093/jac/dkaf314

**Published:** 2025-10-27

**Authors:** Benjamin J Parcell, Carolyne Horner, Rosy Reynolds, Michael Allen, Christopher Longshaw, David M Livermore, Alasdair P MacGowan

**Affiliations:** Division of Population Health and Genomics, School of Medicine, University of Dundee, Ninewells Hospital and Medical School, Dundee DD1 9SY, UK; Department of Medical Microbiology, Ninewells Hospital and Medical School, Dundee DD1 9SY, UK; British Society for Antimicrobial Chemotherapy, 53 Regent Place, Birmingham B1 3NJ, UK; British Society for Antimicrobial Chemotherapy, 53 Regent Place, Birmingham B1 3NJ, UK; Population Health Sciences, University of Bristol, Bristol BS8 2PS, UK; British Society for Antimicrobial Chemotherapy, 53 Regent Place, Birmingham B1 3NJ, UK; MSD Limited, 120 Moorgate, London EC2M 6UR, UK; British Society for Antimicrobial Chemotherapy, 53 Regent Place, Birmingham B1 3NJ, UK; Shionogi B.V., Fifty Paddington, 50 Eastbourne Terrace, Paddington W2 6LG, UK; Norwich Medical School, University of East Anglia, Norwich NR4 7TJ, UK; UK Health Security Agency, Colindale, London NW9 5EQ, UK; Bristol Centre for Antimicrobial Research & Evaluation (BCARE), Severn Infection Sciences, Pathology Sciences Building, North Bristol NHS Trust, Southmead Hospital, Westbury-on-Trym, Bristol BS10 5NB, UK

## Abstract

The BSAC Resistance Surveillance Project ran from 1999 to 2019, amassing an unrivalled collection of almost 100 000 bacterial isolates from bloodstream and lower respiratory tract infections in the UK and Ireland. It was initiated in response to increasing antimicrobial resistance and supplemented existing surveillance schemes, enhancing the understanding of resistance epidemiology by estimating species prevalence within collection groups together with levels of antibacterial resistance, presented in terms of MICs and percentage susceptibility for each species/antibiotic combination tested. Generated data were explored to monitor and identify factors shaping resistance trends, and to profile antibacterial resistance patterns in specific geographies, settings and patient populations. The release of data and/or bacterial isolates led to a rich repository of published peer-reviewed papers. Additionally, the promotion of the BSAC standardized susceptibility testing method resulted in greater uniformity of antimicrobial susceptibility testing in hospital microbiology laboratories. Over time, public health laboratories’ surveillance systems became increasingly comprehensive, and the BSAC Project ceased in 2019. This invaluable collection is now housed in the University of Dundee, in collaboration with the University of St Andrews. We highlight the collection’s unique timeliness, and how the BSAC Project contributed to key interventions for infection prevention and control, public health and antimicrobial stewardship. We demonstrate the utility and benefits of the Project outlining the collection’s future applications as an important bioresource. It comprises well-defined bacterial isolates—many now sequenced—with MIC data and demographic information. This legacy is available to researchers via the Tayside Biorepository and custodian contacts.

## Introduction

Antimicrobial resistance (AMR) rose in the political and media agenda in the early 1990s as it became apparent that ‘superbugs,’ notably MRSA, were increasing and negatively impacting the health of patients.^1^

The proportion of *Staphylococcus aureus* bacteraemia cases due to MRSA rose from 1% to 2% in 1990–1992 to around 40% by 2000 in the United Kingdom and this increase coincided with the emergence of the epidemic strains EMRSA-15 and -16, which rapidly established endemicity in many hospitals, with chains and clusters of nosocomial transmission.^2^ The BSAC identified that existing antimicrobial surveillance was not adequately robust for clinical and public health purposes and, in 1996/7, formed a working party to establish a national surveillance project. Following this, a call came in 1998 from the UK Standing Medical Advisory Committee (SMAC) Sub-Group on AMR to develop an adequately-resourced national surveillance programme to assist in understanding and controlling the spread of resistance.^3^ Previous experience from the Alexander Project (an international, multicentre, longitudinal surveillance study of resistance in common respiratory pathogens) suggested that a similar sentinel surveillance system would be feasible in the UK and Ireland and would bring many benefits.^4^

MacGowan *et al*.,^5^ in this Supplement, provide further background detail of the events that led up to the establishment of the flagship BSAC Resistance Surveillance Project. This Project was initiated to provide surveillance of antibacterial resistance in key clinical pathogens in the UK and Ireland. It ran from 1999 to 2019 during a time of significant change and transformation in terms of public health action for AMR. This paper will discuss some of these key milestones in more detail to highlight the unique timeliness and benefits of its Surveillance Programmes as well as the collection’s current and future value and utility.

During the early 2000s, there was growing political, clinical and media pressure to reduce AMR and infection rates.^6,7^ Key responses included the Health Act 2006 Code of Practice, national infection prevention and control (IPC) directives and initiatives to improve hand hygiene compliance, hospital environment inspections and cleaning of healthcare premises.^8,9^ National evidence-based guidelines were developed to address healthcare-associated infections (HCAIs), including those associated with devices such as urinary and central venous catheters.^8,10^

Mandatory reporting of MRSA bloodstream infection (BSI) was introduced in England in 2001.^11^ Later, in 2004, mandatory reporting was introduced also for *Clostridioides difficile* infection (CDI) involving patients aged 65 years and over.^12^ The CDI surveillance definitions were then expanded in April 2007 to include all cases in patients aged 2 years and over. The aims were to cut MRSA bacteraemia cases by 50% by 2008 and to reduce CDI by 30% by 2010–2011.^13^ Changes also included the introduction of national mandatory screening of all admissions for MRSA in December 2010 by National Health Service (NHS) England.^14^ Centrally-issued penalties were imposed on NHS hospital trusts with excessive numbers of MRSA and CDI cases to highlight that HCAI target failures were the responsibility of the trust board, not just clinicians.^15^

Mandatory *Escherichia coli* bacteraemia surveillance was added in England in June 2011 following observed year-on-year increases; in April 2017, *Klebsiella* spp. and *Pseudomonas aeruginosa* bacteraemias were added to the mandatory surveillance scheme.^12^ With the exception of *S. aureus* and methicillin resistance, these mandatory schemes do not include information on AMR but do measure the burden of invasive disease.

In the same period, there was also increased focus on antimicrobial stewardship, partly to reduce the risk of CDI. A key milestone, in 2003, involved an allocation of 12 million pounds to hospital pharmacists by the UK Department of Health to improve the prescribing of antibiotics within trusts.^16^ The main consequence was the development of a cadre of ‘Antimicrobial Pharmacists’ serving virtually all hospitals in the UK. This led to a raft of actions such as: (i) revision of antimicrobial guidelines to reduce the use of the so-called ‘4C’ antibiotics (ciprofloxacin, clindamycin, cephalosporins and co-amoxiclav) that were especially blamed for selection of CDI; (ii) improved multidisciplinary interactions between Microbiology, Infectious Diseases and Pharmacy teams via ward rounds and meetings, as well as (iii) increased training and education for healthcare professionals on appropriate antimicrobial use.^16^ This, in turn, led to significant shifts in antibiotic prescribing, with successive decreases between 2007 and 2010 in the proportions of patients with CDI in England who had been prescribed either fluoroquinolones or cephalosporins.^11^ Significant and quantifiable reductions in both MRSA bacteraemia and CDI were seen following the implementation of multimodal IPC interventions (including cleaner hospitals and improved care and management of intravascular catheters) and antibiotic stewardship interventions. In addition, the realization that huge outbreaks could lead to costly and embarrassing litigation may have pressured Trusts to reach these targets. By the end of March 2008, the Department of Health reported that the NHS had achieved a dramatic 57% reduction in MRSA BSI and 41% reduction in CDI in England. However, subsequent *E. coli* BSI targets have proved much harder to achieve.^17^

## Establishing an effective surveillance system

The BSAC Resistance Surveillance Project was developed with two separate Programmes, respectively assessing the prevalence of resistance in pathogens frequently implicated in community-associated and (later) hospital-acquired respiratory tract infections, and in bloodstream infections (BSIs). The Respiratory Programme began, in 1999, collecting and centrally testing *Streptococcus pneumoniae*, *Haemophilus influenzae* and *Moraxella catarrhalis* from clinical samples. Following the success of this first Programme, a Bacteraemia Programme was established in 2001 to collect and test the most frequent Gram-positive and -negative pathogens responsible. MacGowan *et al*.^5^ provide further information on the establishment of the Project, and how it was designed to complement and cross-reference with Public Health Laboratory Service (now the UK Health Security Agency (UKHSA)) surveillance, undertaken by collection of hospitals’ own routine susceptibility data for bloodstream isolates.

The BSAC Programmes were designed so that a total of 25–40 collecting laboratories from across the UK and Ireland contributed isolates. These were tested at the UKHSA’s predecessor organisations—the Public Health Laboratory Service (PHLS), the HPA and Public Health England (PHE) in London—or at LGC in Fordham (previously Quotient Bioresearch and GR Micro in London). These ‘Central Laboratories’ undertook identification of the bacterial isolates and measured MICs by the BSAC agar dilution method.^18^ Further testing by various methods such as PCR was carried out to confirm specific antibiotic resistance mechanisms. More information on the methodology can be found in the paper by Allen *et al*. published in this Supplement.^18^

## Dissemination of results and sharing of data

The BSAC Resistance Surveillance Working Party strove to disseminate and report their findings, with first outputs of the Project being published in 2001 as posters at the 22nd International Congress of Chemotherapy (ICC) and 41st Interscience Conference on Antimicrobial Agents and Chemotherapy (ICAAC).^19,20^ Many publications and posters followed, and the BSAC website lists and hosts over 130 of these outputs.^21^ To enable collaborations and public engagement the BSAC surveillance team previously made their antibiotic susceptibility data, including MIC results, open access to researchers, policy makers and the public via the internet. A website (now discontinued) allowed users to create graphs and tables of resistance rates. In addition, the BSAC also offered the research community access to the bacterial isolates for further testing. This became a valuable bioresource, accelerating research into AMR and its molecular epidemiology.

## Utility of the BSAC Resistance Surveillance Project

### Testing of new antibiotic agents

**Figure 1.**
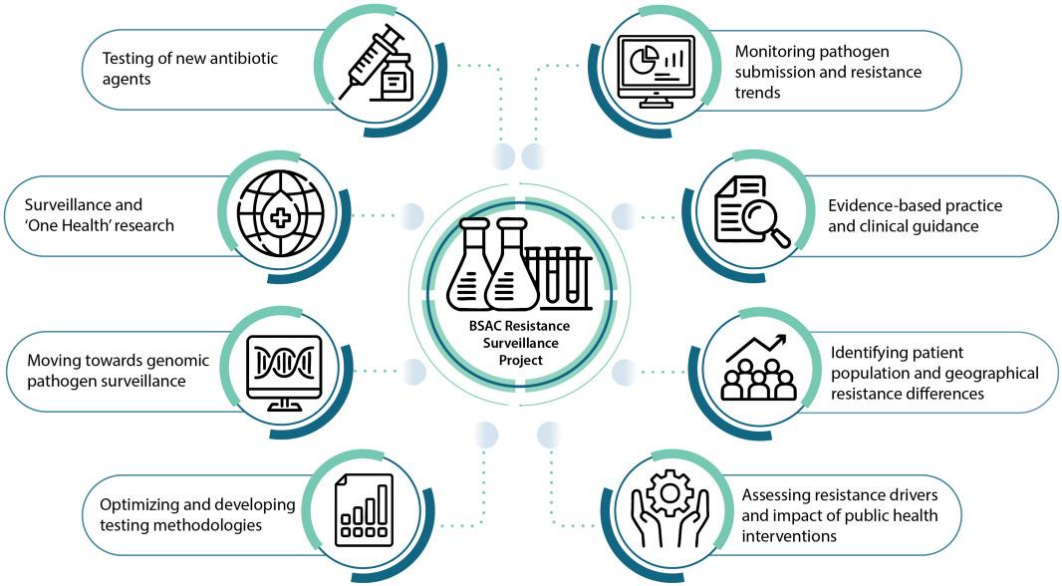
Applications of the BSAC Resistance Surveillance Project.

The Programmes had many different applications (Figure 1). One of these, which also ensured funding, was to test investigational or newly marketed agents against a nationally representative contemporaneous collection of isolates also tested with a core panel of routinely used antibacterials. This approach had several benefits for the Project’s sponsors as it enabled cost-effective testing of their new agent’s performance, along with access to results for the comparator agents tested. Thus, for example, it allowed direct comparison of the two licenced anti-MRSA cephalosporins, ceftaroline and ceftobiprole, with the findings now published.^22^ Company-sponsored studies, by contrast, have typically compared one or other of these molecules with established agents, but without head-to-head comparison.

In addition, there was the added credibility of results being produced independently by a scientific society rather than an individual company or its direct grantees.

### Monitoring pathogen submission and resistance trends

The overarching aim of the Project was to study AMR epidemiology, documenting the prevalence of different species within groups and of antibacterial resistance, with comprehensive MICs and percentage susceptibility for each species/antibiotic combination tested.^3,23^ The data were collected to inform clinical and public health practice.

The BSAC Resistance Surveillance Working Party could review temporal changes in the proportions of different species within collection groups, notably showing the progressive replacement of *Enterococcus faecalis* by *Enterococcus faecium* as the predominant enterococcus species in UK bacteraemias.^24^ Temporal rises in resistance likewise could be tracked, for instance the increase of cephalosporin resistance due to CTX-M extended-spectrum β-lactamases (ESBLs) in *E. coli* from 2002 to 2007 described in the paper by Reynolds *et al*.^25^ These changes were associated with the emergence and proliferation of ST131 *E. coli*. Carbapenem resistance was also monitored and methods used to seek carbapenemase genes can be found in the paper by Allen *et al.*^18^

Additionally, the data could be used to screen for subtle increases in MICs, for instance, vancomycin MIC creep among MRSA (although this was not found).^26^ Such analyses depended on having isolates stored, so that they could be retested over an extended period under exactly the same conditions.

The BSAC Resistance Surveillance Project also highlighted resistances, which were not detected by routine testing: for example, it identified that inherent resistance to colistin in *Enterobacter cloacae* complex was frequent and mostly associated with *Enterobacter asburiae*. Routine monitoring would not have found this because colistin is rarely tested, except against carbapenem-resistant isolates, and because reporting to UKHSA is typically as ‘*E. cloacae* complex’.^27^

### Evidence-based practice and clinical guidance

One important function of the surveillance was to review, using MIC data, the appropriateness of particular antibiotics as empirical treatment for bloodstream and lower respiratory tract infections. This information was used as an evidence base for clinical decision-making through guidance development and publications.^28–30^ It was also used, e.g. to develop a prescribing algorithm for use in conjunction with rapid pathogen identification from nosocomial pneumonia in the INHALE (Potential of Molecular Diagnostics for Hospital-Acquired and Ventilator-Associated Pneumonia in UK Critical Care) multicentre randomized controlled trial.^31^

### Identifying patient population and geographical resistance differences

In some instances, the BSAC team highlighted unusual resistance patterns in certain bacteria from submitting laboratories, or in certain patient populations. Examples include the identification of many *E. faecalis* isolates with high-level resistance to both ciprofloxacin and gentamicin. Typing by pulse-field gel electrophoresis confirmed two large ‘epidemic’ clusters that had become established across various hospitals.^32^ The ability to interrogate the surveillance system data in such detail led to the finding that resistance had emerged in particular demographics, as in the case (before the proliferation of ST131) of ciprofloxacin-resistant *E. coli* in bacteraemias being largely from men.^33^

### Assessing resistance drivers and impact of public health interventions

Surveillance Project data have also been used to explore the relationship between factors that could shape antibiotic resistance, such as antibiotic prescribing. The BSAC Surveillance Working Party used their data along with those from the then Health Protection Agency’s LabBase/CoSurv system and the European Antimicrobial Resistance Surveillance System (EARSS) to investigate associations between trends in penicillin and macrolide resistance amongst pneumococci, making a comparison to antibiotic sales before the widespread deployment of PCV7 (which greatly disrupted the previous epidemiology). Pooled estimates from all datasets indicated significant reduction in penicillin non-susceptibility among pneumococci in the UK, since 1999 (*P* < 0.004), which may have reflected a reduction in community antibiotic use of penicillins. The authors, however, highlighted that no parallel reduction in macrolide resistance had been seen, despite reduced sales. They reasoned that this may be due to the ‘fitness’ of a single clone of serotype 14 V *S. pneumoniae*, designated England 14^–9^, which had been established in the UK since around 1993 and which, by 2005, accounted for ∼60% of all macrolide-resistant invasive isolates.^34^

In a later study, data from both the BSAC surveillance and the UKHSA’s national database were interrogated to both reveal there had been significant increases in cephalosporin and quinolone resistance amongst *Enterobacterales* BSI from 2001 to 2006, with cephalosporin resistance largely due to the spread of CTX-M ESBLs.^35^ Then, from 2007 to 2011, non-susceptibility to cephalosporins and quinolones declined, and it was suggested that this reflected major reductions in the use of cephalosporins and quinolones, implemented primarily to reduce the selection of *C. difficile.* Further detail is given in the paper by Reynolds *et al.*^25^ of this series. ESBL-producing *E. coli* and *K. pneumoniae* continued to increase in continental European countries, where there was not a parallel switch away from cephalosporins and quinolones.

The introduction of vaccines is a public health intervention that can have a major impact on human health. Examples of the impact of vaccines on resistance trends, and on serotype replacement, are described in the paper by Horner *et al*.^36^ of this series, which covers surveillance data for respiratory and invasive *S. pneumoniae* across the introduction of pneumococcal conjugate vaccines, PCV7 and 13.

### Optimizing and developing testing methodologies

Data from the Surveillance Project have served to warn microbiologists about testing issues, particularly in respect of ESBL detection. Specifically, warnings were raised to underscore the potential for false-positive ESBL results when testing *Klebsiella oxytoca* strains that hyperproduce K1 β-lactamase.^37^ The authors warned that this risk is magnified if laboratories test only a few β-lactam antibiotics whilst solely using cephalosporin-clavulanate ‘ESBL tests’ to define ESBL production.

In another example, BSAC MIC data supported the development of an algorithm to predict resistance from whole-genome sequences of *E. faecium*.^38^ In this study, researchers created a bespoke catalogue of 228 genetic markers for resistance for 12 antibiotics that could be used to treat *E. faecium* infections. The authors then used the BSAC-determined antibiotic susceptibility phenotypes of the isolates to validate the diagnostic accuracy of this catalogue. They found that they could achieve near-complete genotype–phenotype concordance for ampicillin, ciprofloxacin, vancomycin and linezolid, with high statistical sensitivity for tetracycline, teicoplanin and high-level aminoglycoside resistance.

### Moving towards genomic pathogen surveillance

To date, over 4400 BSAC isolates have been sequenced by the Wellcome Sanger Institute, for various studies. These include 349 Group A *Streptococcus*, 80 *Pseudomonas* spp., 636 *Enterococcus* spp., 1098 *E. coli*, 1282 multidrug-resistant (MDR) Gram-negative isolates (including *Acinetobacter* spp.*, Enterobacter* spp.*, E. coli, Klebsiella* spp. and *Serratia* spp.), along with over 989 *S. aureus*.^39–43^ Notably, some of these studies have employed genomic surveillance to characterize bacterial populations over large geographic and temporal scales.

In one study, researchers sequenced 168 *E. faecalis* isolates from three collections (National Collection of Type Cultures, BSAC and Cambridge University Hospitals NHS Foundation Trust (CUH)) to carry out a genome-based characterization of hospital-adapted bloodstream infection *E. faecalis* lineages.^44^ They found that three lineages (termed L1, L2 and L3) predominated, with 53% of isolates clustering into these. L1 was demonstrated in each year of the collection; however, L2 was mostly identified between 2001 and 2006, with only two subsequent representatives found after that time. The authors postulated that this could suggest clonal replacement, much like the phenomenon observed for MRSA. After reviewing geographical sources, they considered L1 and L2 to be epidemic clones as they were nationally distributed, whereas L3 was only isolated in two locations (CUH and a hospital in the East Midlands referral network). The researchers found that putative virulence and antibiotic resistance genes, including vancomycin, were over-represented in L1, L2, and L3 isolates combined compared to the remainder lineages.

In another study, authors at the Sanger Institute sequenced 1013 UK bloodstream MRSA isolates submitted to the BSAC by 46 laboratories between 2001 and 2010.^39^ From this, they produced an overview of the population structure of MRSA in the UK, describing how the majority of isolates were clonal complex 22 (CC22-MRSA), also known as UK EMRSA-15. They then mined these data and identified differences in local and regional antibiotic resistance profiles. Phylogenetic reconstruction was carried out on the CC22 isolates. The authors placed the genomes from two confirmed outbreaks and a pseudo-outbreak (where epidemiology suggested an MRSA cross-infection but whole-genome sequencing demonstrated unequivocally that the isolates were unrelated) into the CC22 phylogenetic tree and found that there was a clear demarcation between the two outbreak clusters whilst isolates from the pseudo-outbreak were scattered throughout the tree.^45^ Thus, the genetic database could be used to investigate suspected outbreaks.^39^

The genomic sequencing data of MRSA from the BSAC Resistance Surveillance Project supported a further analysis by the Sanger Institute, looking at the population structure of bloodstream EMRSA-15/CC22 between 1998 and 2012 in relation to regional patterns of patient transfers. Researchers used isolates that had been submitted to 52 hospitals in the UK and Ireland. The authors identified distinct bacterial population structures that mirrored each hierarchical level of the referral networks (from hospitals, to regions, to countries) and identified that patient referrals played a key role in EMRSA-15 transmission.^40^

Numerous BSAC isolates have undergone sequencing as part of other studies. As mentioned earlier in this manuscript, an epidemiological investigation [including sequencing and biochemical analysis of lipopolysaccharides (LPS)] was carried out to examine the first 50 colistin-resistant *E. cloacae* complex isolates received by the BSAC Resistance Surveillance Project in comparison with an equal number of susceptible isolates.^27^ The study identified that colistin resistance was increasing amongst BSI isolates and was particularly associated with *E. asburiae*. Resistant isolates were widely scattered in time and place amongst submission sites and the BSAC team concluded that the resistance was a frequent inherent trait in some genogroups. Biochemical analysis revealed that resistant and hetero-resistant isolates exhibited more complex lipid A patterns compared with susceptible isolates specifically, forms with 2-hydroxylation and palmitoylation (typically generated by LpxO and PagP enzymes) were identified, and membranes rich in these may be less susceptible to disorganisation by polymyxins. None of the sequenced isolates contained *mcr* genes.

### Surveillance and ‘one health’ research

‘One Health’ is an integrated, unifying approach, which aims to balance and optimize the health of people, animals and the environment. The BSAC Resistance Surveillance Project supported AMR research in this area by enabling researchers to examine the genetic relatedness of bacteria from human, animal and environmental origins.

In one study, researchers compared 423 vancomycin-resistant *E. faecium* in municipal wastewater treatment plants with 187 bloodstream vancomycin-resistant *E. faecium* from five hospitals in the East of England, with some of the latter isolates from the BSAC surveillance.^41^ Phylogenetic analysis revealed widespread distribution of hospital-adapted vancomycin-resistant *E. faecium* beyond acute healthcare settings and into the environment.

In a separate study, the same researchers used pathogen genomics to investigate whether livestock could be a potential reservoir for *E. coli* strains that later cause human BSI.^42^ The researchers sequenced 431 *E. coli* isolates from livestock farms and retail meat in the East of England and compared these with the genomes of 1517 *E. coli* bacteria associated with human BSI from the BSAC Project. Phylogenetic core genome comparisons revealed that the livestock and human BSI isolates were genetically distinct. In addition, the researchers used long-read sequencing to analyse mobile genetic elements and identified that the prevalence of shared AMR genes between livestock and human isolates was low. These results, taken together, suggest that the *E. coli* responsible for human BSI are very unlikely to originate from livestock. This entirely accords with separate work, not predicated on the BSAC collections, showing that ST131 is the predominant ESBL-producing and fluoroquinolone resistant *E. coli* lineage from human BSI and gut colonisations but is extremely rare or absent among ESBL *E. coli* from foodstuffs.^46^

## Closure of the BSAC Resistance Surveillance Project

The BSAC Resistance Surveillance Project assembled a collection of ∼100 000 bacterial isolates (with associated resistance data) from bloodstream and lower respiratory tract infections over two decades until its closure in 2019, as described by Allen *et al.*^18^ in this series.

This closure reflected multiple pressures, including financial constraints on the pharmaceutical funders, with fewer antibiotics being developed by profit-making companies, increased takeovers within the industry and contracts ending. In addition, UKHSA surveillance has improved, with more antibiotic resistance data reported under the voluntary surveillance for bacteraemia isolates than 20 years ago. Moreover, these data have improved in quality, reflecting wide adoption of MALDI-TOF mass spectrometry for identification and better standardized susceptibility testing by BSAC and, latterly, European Committee on Antimicrobial Susceptibility Testing (EUCAST) methods.

Research into pathogen genomics is gaining traction. Significant investment in sequencing facilities at reference laboratories occurred in response to the COVID-19 pandemic. In parallel, several ‘national pathogen genomics strategies’ were introduced across the UK, paving the way for greater use of sequencing for surveillance and the investigation of infectious public health threats.^47–49^ Time will tell how far these will be expanded to include resistant pathogens.

## Future utility as a bioresource

After the official closure of the BSAC Resistance Surveillance Project, a call was made by the Society to find a new home for the isolate collection. After competitive application, the University of Dundee, in collaboration with the University of St Andrews, was successful. Both universities are at the forefront of AMR research, with international experts working on sequencing, diagnostics, vaccine development and therapies, including new antibiotics, combination therapy and repurposing of antimicrobial agents.

Since relocation to Dundee, the collection has been maintained at −80°C, with ongoing local and international interest from researchers to access resistance data and bacterial isolates. Bacterial isolates and data can be sourced via applications to the Tayside Biorepository part of the NHS Research Scotland (NRS) Biorepository Network (https://nrsbiorepository.hicservices.dundee.ac.uk/), which provides governance support through a robust review process of applications (See Box 1 for contact details).

Box 1. **Custodian contacts for supply of isolates and data**

**Dr Benjamin J. Parcell**, Clinical Senior Lecturer and Honorary Consultant Microbiologist, Division of Population Health and Genomics, School of Medicine, University of Dundee, Ninewells Hospital and Medical School, Dundee, DD1 9SY, United Kingdom. Email: bjparcell@dundee.ac.uk or benjamin.parcell@nhs.scot.

**Stuart Reid**, Senior Healthcare Scientist at Ninewells Hospital and Medical School, NHS Tayside. Email: stuart.reid5@nhs.scot.

The re-housed collection is already supporting several research studies. These are varied, as exemplified below, ranging from wet-lab focused work to big data analysis. Some projects are newly initiated whilst, in others, research teams further explore previous research findings. One of the first applications for isolates came from a group who are investigating and exploring the structural diversity of enterococcal polysaccharide antigen. As part of this, they are building on their previous findings, in which they identified that this polysaccharide is involved in AMR and virulence.^50^ Another application, from a different group, aims to examine numbers of BSAC isolates using a newly-developed ‘rapid tolerance screen’ in order to identify bacterial populations that can survive transient exposures to an otherwise lethal concentration of antibiotic, without exhibiting resistance in terms of raised MICs^51^ Other research teams continue to characterize the BSAC Group A streptococci isolates, which were sequenced previously.^52^ Part of this involves wet lab work to investigate the microorganisms’ ability to form biofilms and survive in whole human blood or on human tonsil tissue and their adherence and invasion of human cell lines. The group is also looking at resistance to opsonophagocytosis and the expression levels of virulence factors. In addition, the collection has also been used for a study exploring competitive interactions between bacteria in relation to Type VI secretion systems (T6SS). Some bacteria, notably *Serratia marcescens*, use these to deliver antibacterial toxins directly into neighbouring competitor bacteria, killing or disabling them.

Other studies, demonstrating the diverse application of the collection, include one on the use of machine learning to predict antibiotic resistance, and another—recently funded—to investigate potential antibiotic adjuvant therapies for the treatment of recalcitrant *P. aeruginosa* infections.

Interrogation of already-generated sequencing data continues. One example of this involves a recent genomics-based molecular epidemiology study using longitudinal sequence and resistance data for *E. coli* from BSI to investigate the contribution of antibiotic use to clonal success in the UK and Norway, *vis-a-vis* intra-strain competition for colonization and infection.^53^ The authors found that the resistance profiles (in particular to trimethoprim) of successful clones varied and did not always correlate with, nor reflect, patterns of antibiotic usage. They suggest that further research should be carried out to understand the effects of antibiotic selection, with additional focus on non-antibiotic-related factors that may influence colonization competition and clonal success.

The BSAC collection has also recently supported research into developing an *in-silico E. coli* capsular typing database to quantify K-antigen-related invasive potential across different extra-intestinal pathogenic *E. coli* lineages. The researchers found that several K-types and lineages contributed disproportionately to invasive extra-intestinal pathogenic *E. coli* disease.^54^ They postulate that these findings could be used to improve extra-intestinal pathogenic *E. coli* diagnostics and inform predictive regional risk maps.

These examples underscore the breadth of research opportunities the collection provides and the scope to return to its unique data, building on the Society’s initial work.

## Summary

During its active years, the BSAC Resistance Surveillance Project provided an elegant contribution to resistance surveillance, enmeshing with UK systems that collected large-scale but less-rich data, as well as bringing many additional benefits across research and clinical practice. The collection represents a ‘time capsule’ representing trends in antibiotic resistance that occurred during a period of great transformation involving public health action to reduce AMR, HCAI and, through vaccination, invasive pneumococcal disease.

The beauty of the collection is that it was assembled according to a defined surveillance protocol using standardized antibiotic testing to measure MICs. Many other surveillance projects are single-centre or *ad hoc* special-interest collections or have too few centres per country to be representative. By contrast, the BSAC Project collected isolates from a large number of clinical centres across the British Isles, precluding overrepresentation of outbreaks affecting one or two sites. The collection contains highly resistant strains and organisms of interest. The legacy of the collection continues to live on as an important bioresource—and this maintenance is very much down to the meticulous care and planning taken by the original founders, many of whom remained associated with the Project throughout its lifetime.

There is great potential for researchers to continue to use the collection to unlock the secrets of AMR for many years to come. Access to the isolates is available to researchers via the Tayside biorepository and via the custodians listed in this paper (Box 1). It is a rich resource of well-described bacterial isolates, with MIC-based resistance data and some anonymized demographic information. The significant and growing number of whole genome sequences that are available will allow further investigation into resistance, toxins, virulence and pathogenic factors and mobile genetic elements. As bacteria and resistance continue to evolve it will, increasingly, become a historic reference collection from around the turn of the century. In short, the collection remains relevant and important into the future, as we deepen our understanding of antibiotic resistance and develop new strategies to combat it.

## Transparency declarations

B.J.P.: consultancy: Genpax. C.H. was employed by Una Health Limited and QuantuMDx Group Limited during the period of data analysis and manuscript preparation. MA is a Trustee of the BSAC Council and is employed by MSD (UK) Limited, London, UK. MA has shareholdings in Merck & Co., Inc., Rahway, NJ, USA. C.L. is a Trustee of the BSAC Council and is employed by Shionogi B.V. DML: Advisory Boards or ad hoc consultancy: AdjuTec, AstraZeneca, Centauri, GenPax, GSK, Lipovation, Meiji, Paion, Shionogi, Sumitovant, Thermo Fisher and Wockhardt; Paid lectures – bioMérieux, Pfizer, Shionogi and Zuellig Pharma. Relevant shareholdings or options – GenPax, GSK, Merck & Co Inc. and Revvity amounting to less than 10% of portfolio value. DML also has shareholdings in Arecor, Genincode, Probiotix, Rua Life Sciences, SkinBiotherapeutics and VericiDx (all with research/products pertinent to medicines or diagnostics) through Enterprise Investment Schemes, but has no authority to trade these holdings directly. A.P.M. holds research grants/activities with Merck, Shionogi, InfectoPharm, GSK, Roche, BioVersys, VenatoRx Pharmaceuticals, iFAST, Oxford Drug Design, JPIAMR and NIHR; and provides consultancy advice to Shionogi, Roche, Bicycle Therapeutics and BioVersys. All other authors have nothing to declare.
